# The pediatric supratentorial MYCN-amplified high-grade gliomas methylation class presents the same radiological, histopathological and molecular features as their pontine counterparts

**DOI:** 10.1186/s40478-020-00974-x

**Published:** 2020-07-09

**Authors:** A. Tauziède-Espariat, M-A Debily, D. Castel, J. Grill, S. Puget, A. Roux, R. Saffroy, M. Pagès, A. Gareton, F. Chrétien, E. Lechapt, V. Dangouloff-Ros, N. Boddaert, P. Varlet

**Affiliations:** 1 Department of Neuropathology, GHU Paris-Psychiatrie et Neurosciences, Sainte-Anne Hospital, 75014 Paris, France; 2 U981, Molecular Predictors and New Targets in Oncology, INSERM, Gustave Roussy, Université Paris-Saclay, 94805 Villejuif, France; 3 Univ. Evry, Université Paris-Saclay, 91000 Evry, France; 4 Département de Cancérologie de l’Enfant et de l’Adolescent, Gustave Roussy, Université Paris-Saclay, 94805 Villejuif, France; 5 Department of Pediatric Neurosurgery, Necker Hospital, APHP, Université Paris Descartes, Sorbonne Paris Cite, 75015 Paris, France; 6 Department of Neurosurgery, GHU Paris-Psychiatrie et Neurosciences, Sainte-Anne Hospital, 75014 Paris, France; 7 Department of Biochemistry and Oncogenetic, Paul Brousse Hospital, 94804 Villejuif, France; 8 Equipe SiRIC RTOP Recherche Translationelle en Oncologie Pédiatrique, Institut Curie, Paris, France; 9 INSERM U830, Laboratoire de Génétique et Biologie des Cancers, Institut Curie, Paris, France; 10 SIREDO: Care, Innovation and Research for Children, Adolescents and Young Adults with Cancer, Institut Curie, Paris, France; 11 Paediatric Radiology Department, Hôpital Necker Enfants Malades, AP-HP, University de Paris, INSERM U1163, Institut Imagine, Paris, France

Recent genomic and epigenomic analyses have pointed out the heterogeneity of tumors from a same histopathological group and have identified key oncogenic alterations that enabled the description of novel tumor entities. Thus, pediatric high-grade gliomas (HGG) comprise a heterogeneous group of tumors, including H3 K27M-mutant, H3 G34-mutant, *IDH-*mutant and H3/*IDH*-wildtype HGG. Furthermore, the H3/*IDH*-wildtype HGG group has recently been divided into three molecular entities based on their DNA methylation profile: Receptor tyrosine kinase type I (RTK I) and II (RTKII), and *MYCN*-amplified, the latter representing the most frequent subgroup (41% of cases, 36/87) [4]. However, the current 2016 WHO classification does not discriminate between them. In addition, data on these entities came from large series collectively deciphering molecular landscape of HGG. Therefore, the HGG-MYCN subgroup remains poorly characterized and clinical, imaging and pathological data are scarce (Supplementary Table S1).

We investigated data from five pediatric supratentorial HGG-MYCN diagnosed by DNA methylation profiling at our institution (one case included in [7]) and we pooled them with methylation class pediatric HGG-MYCN of the literature (*n* = 59) [4, 5, 7]. Therefore, we analyzed clinical, histopathological and molecular data of pediatric supratentorial HGG-MYCN and compared them to their pontine counterparts [9] and did a systematic review of four groups of supratentorial pediatric HGG (including 62 H3 K27M-mutant gliomas, 31 H3-G34 mutant gliomas, 44 HGG-RTKI and 16 HGG-RTKII) [1–8, 10].

Clinical data of our cases are summarized in Table S2. The median age of pediatric HGG-MYCN (published cases and our own) was 9.0 years (range from 2 to 18) which was lower than H3 K27M (11.0 years) and H3 G34-mutant (13.0 years), RTKI (10.0 years) and RTKII subgroups (10.0 years) [1–8, 10]. This difference was only significant between HGG-MYCN and H3-G34 mutant gliomas (*p* < 0.001) [4, 5, 7, 8]. The sex ratio male/female for HGG-MYCN was 1.3 and 3.4, 0.6, 1.2 and 1.3 respectively for H3 G34-mutant gliomas, H3 K27M-mutant gliomas, HGG-RTKI and RTK2 (but without significant difference) [1–8, 10]. HGG-MYCN were mostly located in the hemispheres (31/37 cases with available data, 83.8%), but 5 (13.5%) were thalamic and one arose from the sellar area [4, 5, 7]. There was a slight predilection for temporal lobes (16/37 cases, 43.2%), which was significantly higher than in other subgroups (*p* < 0.001).

By imaging, no calcifications were observed and only one tumor was hemorrhagic (Case 5). They were well-circumscribed with meningeal attachment (except for thalamic tumors). They appeared as solid hypercellular masses with a restricted apparent diffusion coefficient (ADC) in the main part of the tumors. They displayed slight peri-lesional edema and homogeneous enhancement after contrast injection (Fig. 1). These imaging characteristics were quite similar to their pontine counterparts [9].

**Fig. 1:**
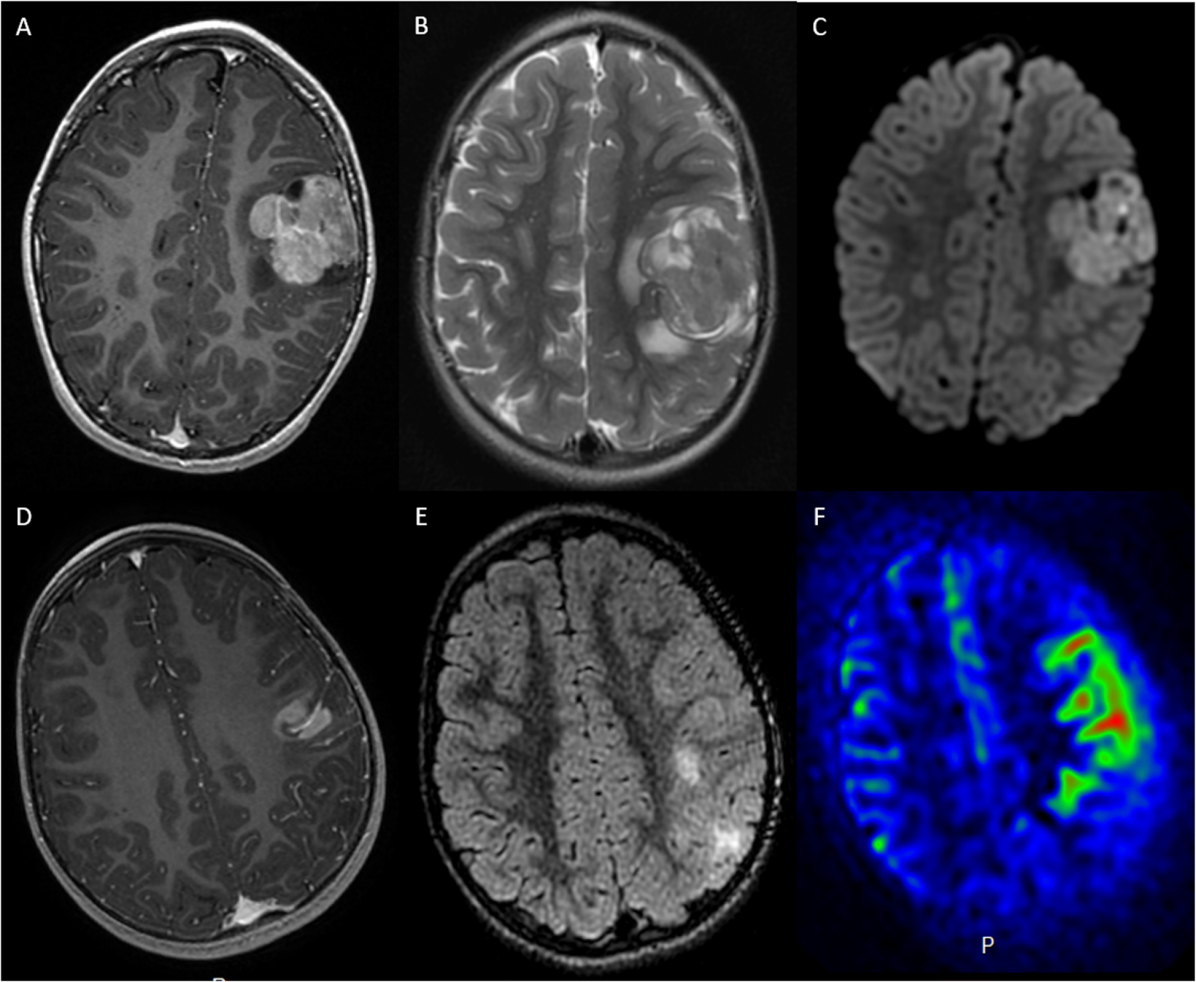
Radiological features of two supratentorial HGG-MYCN. First line: Case 3. (**a**) T1-weighted images after contrast media injection, (**b**) T2-weighted images, and (**c**) diffusion-weighted images: a solid lesion with peri-lesional edema, homogeneous enhancement and hypercellularity (apparent diffusion coefficient (ADC) on diffusion weighted images is restricted in the main part of the tumor). Second line: Case 1. (**d**) T1-weighted images after contrast media injection, (**e**) FLAIR-weighted images and (**f**) cerebral blood flow map using arterial spin labeling: a solid and infiltrative lesion with homogeneous enhancement and high cerebral blood flow

The mean/median progression-free survival was 9.3/9.0 months for HGG-MYCN, 8.8/9.0 months for HGG-RTKI, 8.7/6.3 months for supratentorial H3 K27M-mutant HGG and 10.5/10.0 months for H3 G34-mutant HGG without significant differences in univariate analysis (*p* = 0.421) (Fig. 2) [1–3, 5–8, 10]. The mean/median overall survival (OS) was 16.4/16.5 months for HGG-MYCN, 12.0/11.5 months for HGG-RTKI, 13.9/12.0 months for supratentorial HGG-K27M and 17.6/15.0 months for HGG-G34 without significant differences in univariate analysis (*p* = 0.109) (Fig. 2) [1–3, 5–8, 10]. This median OS was significantly longer (*p* < 0.001) than pontine HGG-MYCN (median OS of 1.5 months, likely due to tumor location) [7, 9].

**Fig. 2:**
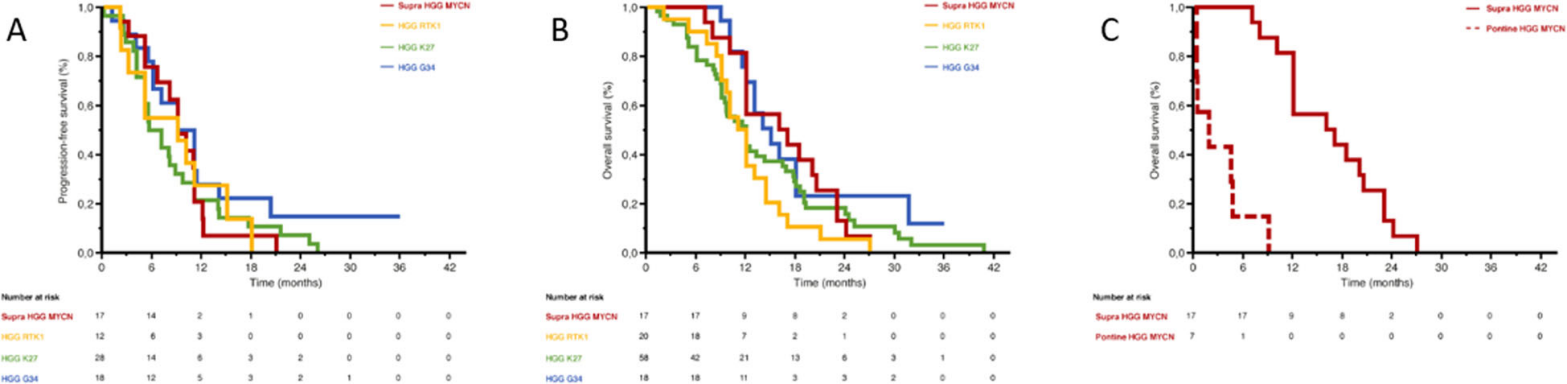
Results of the systematic review of supratentorial molecular subgroups of pediatric HGG. **a** There was no significant difference in terms of progression-free survival (PFS) between HGG-MYCN, HGG-RTKI, supratentorial H3 K27M-mutant HGG and H3 G34-mutant HGG in univariate analysis (*p* = 0.421). **b** There was no significant difference in terms of progression-free survival (PFS) between HGG-MYCN, HGG-RTKI, supratentorial H3 K27M-mutant HGG and H3 G34-mutant HGG in univariate analysis (*p* = 0.109). **c** There was a significant difference in terms of overall survival (OS) between supratentorial HGG-MYCN and pontine HGG-MYCN in univariate analysis (*p* < 0.001)

Histopathological features of all our HGG-MYCN were similar to those described in pontine HGG-MYCN [9]. These undifferentiated neoplasms presented circumscribed nodules and isolated tumoral cells infiltrating the brain (corresponding to the radiological peri-lesional edema). Leptomeningeal extension was common, (Fig. 3a and b). The proliferations were highly cellular, composed of alternating spindle and epithelioid cells with prominent nucleoli (Fig. 3c-e). In all five cases, malignancy was obvious with high mitotic count and proliferation index (mean MIB1 index 66%), necrosis, and microvascular proliferation (Fig. 3d-f). Immunohistochemical findings are summarized in supplementary Table S3. There was no expression of H3K27M, IDH1R132H, Lin28A and a preserved expression of H3K27me3, INI1 and ATRX in all tumors. Tumor cells co-expressed at least one glial and one neuronal marker (Fig. 3g-i). All these results were in line with the literature (20/25 reported cases were initially diagnosed as primary neuroepithelial tumors –PNET) [7]. Contrarily to pontine tumors, no pluriphenotypic pattern was observed in the supratentorial location [9]. In all 5 cases, tumor cells exhibited a strong nuclear and diffuse accumulation of p53 (Fig. 3j) and *TP53* mutations were found by next-generation sequencing analyses (as in 56.2% of reported cases) [5, 7]. Interestingly, loss of PTEN expression was constantly observed in all 5 supratentorial HGG-MYCN (Fig. 3k), contrarily to their pontine counterparts [9]. Tumor cells presented a preserved expression of ATRX in all 5 cases which was consistent with the reported data (25/26) [4]. No *hTERT* promoter mutation was observed in the 5 tumors diagnosed at our institution (possibly due to the small size of our series), contrary to 18.7% (6/32) of reported cases [4, 5].

**Fig. 3:**
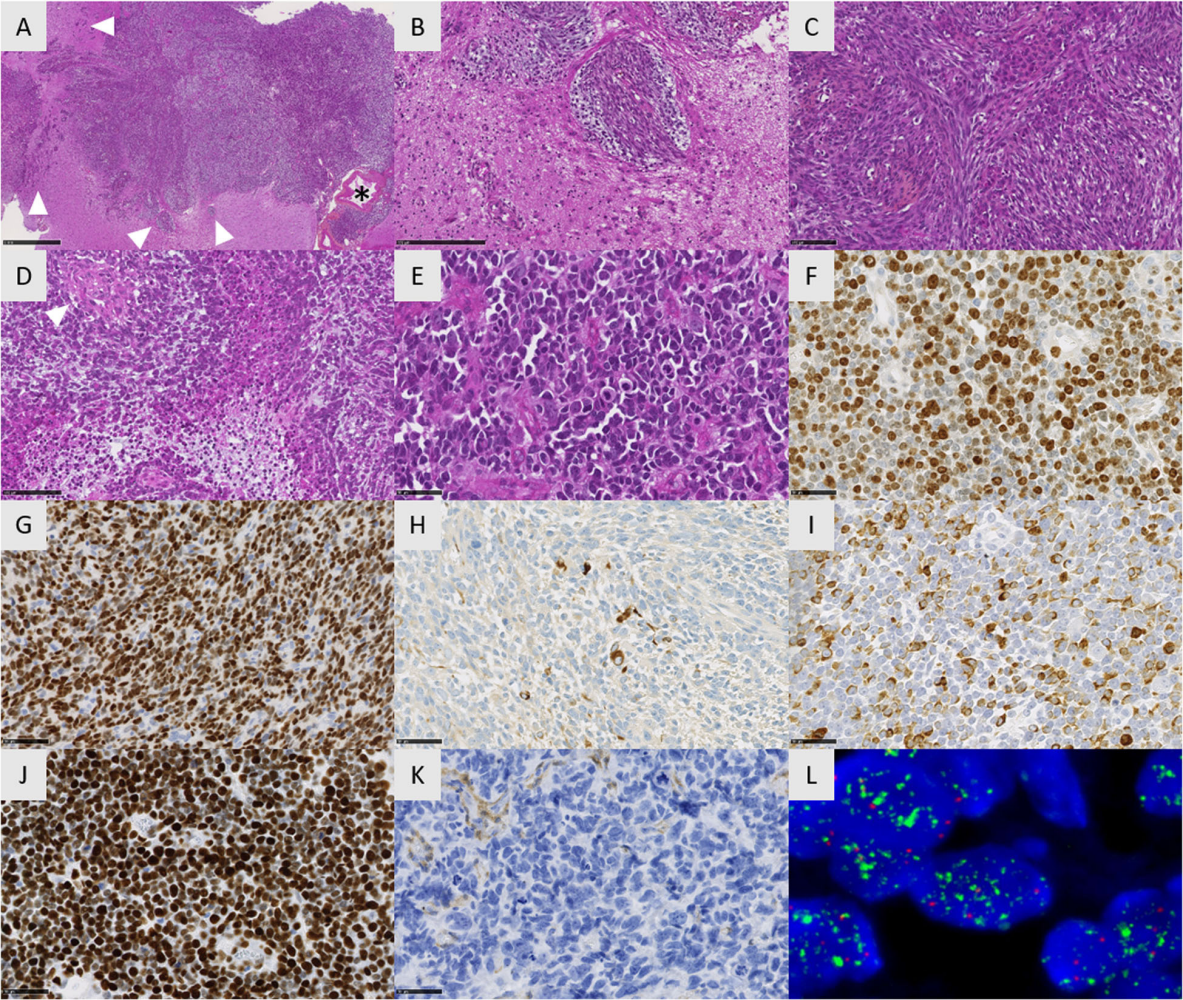
Histomolecular features of HGG-MYCN. **a** Diffuse and solid proliferation with several nodules infiltrating the brain parenchyma (arrowheads) and the leptomeninge with large vessels (asterisk) (Case 2, HPS, × 100 magnification). **b** Dense proliferation of tumour cells organized in nodules following Virchow-Robin spaces around capillaries (Case 2, HPS, × 250 magnification). **c** Highly cellular and undifferenciated proliferation composed of alternating fascicles and nodules (Case 2, HPS, × 250 magnification). **d** Highly malignant tumor with microvascular proliferation (arrowhead) and necrosis (Case 2, HPS, × 400 magnification). **e** Embryonal proliferation composed of hyperchromatic cells presenting anisocaryotic nuclei with numerous apoptotic bodies (Case 3, HPS, × 400 magnification). **f** Elevated proliferation index (Case 2, MIB, × 400 magnification). **g** Diffuse expression of Olig2 (Case 2, × 400 magnification). **h** Focal expression of GFAP by tumor cells (Case 2, × 400 magnification). **i** Expression of neurofilament in numerous tumor cells (Case 3, × 400 magnification). **j** Nuclear accumulation of p53 (Case 2, × 400 magnification). **k** PTEN loss of expression in tumor cells (endothelial cells as positive internal controls). **l** High-level of MYCN amplification by FISH analysis with *MYCN* locus in green signals and control centromeric in red signals (Case 4). Black scale bars represent 1 mm (**a**), 100 μm (**b**) and 50 μm (C to K)

HGG-MYCN is a DNA methylation defined tumor entity based on clustering analyses, and tumors constituting this methylation cluster often exhibit *MYCN* amplification (52.3% of reported cases, 34/65) [4, 7]. *MYCN* amplification is easily detectable by FISH analysis and was observed in the 5 tumors diagnosed at our institution (Fig. 3l). *ID2* amplification is frequently observed in this DNA methylation cluster (72.2%, 26/36 reported HGG-MYCN, including the 5 tumors from our institution), and was reported in one tumor lacking *MYCN* amplification (1/63 cases, 1.6%) [4, 7] suggesting that *ID2* amplification is characteristic of this methylation class and might help diagnose this entity.

Here, we extend the knowledge of pediatric supratentorial HGG-MYCN and present their clinico-radiological and morpho-immunophenotypes. We recommend systematically adding *MYCN* and *ID2* analyses to the diagnostic molecular panel for pediatric H3/*IDH*-wildtype malignant supratentorial tumors with glioneuronal phenotype. Nevertheless, considering the relatively high proportion of tumors belonging to this cluster lacking *MYCN* amplification, this diagnosis can only be made with certainty by DNA methylation profiling and further investigations are needed to better characterize this entity and identify alternative oncogenic drivers to *MYCN* amplification.

## Supplementary information

**Additional file 1: Table S1**. Summary of available data concerning pediatric HGG-MYCN in the literature.

**Additional file 2: Table S2**. Clinical data of pediatric HGG-MYCN of our series.

**Additional file 3: Table S3.** Immunohistochemical profile and molecular data of pediatric HGG-MYCN of our series.
