# Supplementary material for: The pediatric supratentorial MYCN-amplified high-grade gliomas methylation class presents the same radiological, histopathological and molecular features as their pontine counterparts
Source: Acta Neuropathol Commun. 2020 Jul 9;8:104. doi: 10.1186/s40478-020-00974-x (PMC7346460; doi:10.1186/s40478-020-00974-x)
Supplement: Supplementary file 1 — Additional file 1: Table S1. Summary of available data concerning pediatric HGG-MYCN in the literature. [file 40478_2020_974_MOESM1_ESM.docx]

**Table S1. Summary of available data concerning pediatric HGG-MYCN in the literature**

| Data  Study | Cases (n) | Clinical data | Radiological data | Histopathological data | IHC data | Molecular data | | Prognostic data |
| --- | --- | --- | --- | --- | --- | --- | --- | --- |
|  |  |  |  |  |  | DNA-methylation | *MYCN*/*ID2* status |  |
| Korshunov *et al.* | 28 | Incomplete (age and sex for all cases; location not detailed -supratentorial *vs* thalamic- for all cases; no therapeutic data ) | NA | NA | NA | Yes | Yes | Incomplete (no PFS avalaible; median OS without patients’ details) |
| Sturm *et al.* | 25 | Incomplete (age, sex and location for all cases; no therapeutic data) | NA | NA | NA | Yes | Incomplete (no detailed status according to the location supratentorial *vs* brainstem of HGG-MYCN) | Incomplete (PFS and OS for 6 cases) |
| Mackay *et al.* | 6 | Complete (age, sex, location and therapeutic data for all cases) | NA | NA | NA | Yes | Incomplete (*MYCN* status for all cases; no *ID2* status) | Incomplete (no precision about type of recurrence and status of patients at the end of follow-up) |

IHC: immunohistochemical; NA: not available; OS: overall survival; PFS: progression-free survival.
