# Supplementary material for: The pediatric supratentorial MYCN-amplified high-grade gliomas methylation class presents the same radiological, histopathological and molecular features as their pontine counterparts
Source: Acta Neuropathol Commun. 2020 Jul 9;8:104. doi: 10.1186/s40478-020-00974-x (PMC7346460; doi:10.1186/s40478-020-00974-x)
Supplement: Supplementary file 2 — Additional file 2: Table S2. Clinical data of pediatric HGG-MYCN of our series. [file 40478_2020_974_MOESM2_ESM.docx]

**Table S2. Clinical data of pediatric HGG-MYCN of our series**

| Case | Age (YO), sex | Location | Treatment | Recurrence, PFS (months) | Status, OS (months) |
| --- | --- | --- | --- | --- | --- |
| 1 | 3.4, M | Left frontal lobe | PR + CT + RT | Local, 12.1 | D, 18.4 |
| 2 | 3.2, F | Right thalamus | STR + CT + RT | Local, 6.5 | D, 7.9 |
| 3 | 3.4, M | Left fronto-parietal lobe | TR + CT + RT | Local, 12.0 | D, 20.5 |
| 4 | 5.8, F | Left parietal lobe | TR + CT + RT | Local, 10.9 | D, 24.1 |
| 5 | 5.2, M | Right thalamic | B + CT + RT | No (stable disease) | A, 4.0 |

A: alive; B: biopsy; CT: chemotherapy; D: dead; F: female; M: male; OS: overall survival; PFS: progression-free survival; PR: partial resection; RT: radiotherapy; STR: subtotal resection; TR: total resection; YO: year-old.
