# Supplementary material for: The pediatric supratentorial MYCN-amplified high-grade gliomas methylation class presents the same radiological, histopathological and molecular features as their pontine counterparts
Source: Acta Neuropathol Commun. 2020 Jul 9;8:104. doi: 10.1186/s40478-020-00974-x (PMC7346460; doi:10.1186/s40478-020-00974-x)
Supplement: Supplementary file 3 — Additional file 3: Table S3. Immunohistochemical profile and molecular data of pediatric HGG-MYCN of our series. [file 40478_2020_974_MOESM3_ESM.docx]

**Table S3. Immunohistochemical profile and molecular data of pediatric HGG-MYCN of our series**

| Case | Olig2 | GFAP | CD56 | Vim. | NeuN | Syn. | Chr. | NF70 | p53 | EGFR | PTEN | PCK | MIB (%) | *MYCN/ID2* amplifications | NGS alterations |
| --- | --- | --- | --- | --- | --- | --- | --- | --- | --- | --- | --- | --- | --- | --- | --- |
| 1 | 0 | + | + | +++ | 0 | 0 | + | ++ | +++ | + | L | 0 | 90 | Present | *TP53* mutation |
| 2 | +++ | + | + | +++ | + | 0 | 0 | ++ | +++ | + | L | 0 | 90 | Present | *TP53* mutation |
| 3 | +++ | + | ++ | +++ | 0 | + | 0 | ++ | +++ | ++ | NI | 0 | 60 | Present | *TP53* mutation, *PIK3CA* mutation |
| 4 | +++ | ++ | +++ | +++ | 0 | + | 0 | ++ | +++ | +++ | L | 0 | 60 | Present | *TP53* mutation |
| 5 | ++++ | +++ | +++ | +++ | 0 | ++ | 0 | 0 | +++ | +++ | L | 0 | 30 | Present | *TP53* mutation, *EGFR* amplification |

Chr.: chromogranin A; L: loss of expression; NGS: next-generation sequencing; NI: not informative; PCK: pancytokeratin; Syn.: synaptophysin; Vim.: vimentin; +: focal expression; ++: partial expression; +++: diffuse expression.
